# Concentric needle jitter: Reference values in stimulated *Tibialis Anterior* muscle

**DOI:** 10.3389/fneur.2022.957174

**Published:** 2022-07-22

**Authors:** João Aris Kouyoumdjian, Carla Renata Graca

**Affiliations:** Neuromuscular Investigation Laboratory, Department of Neurological Sciences, Psychiatry and Medical Psychology, State Medical School (FAMERP), São Paulo, Brazil

**Keywords:** single-fiber electromyography, jitter, concentric needle electrode, *Tibialis Anterior* muscle, electrical activation, myasthenia gravis

## Abstract

Calculating the reference values for jitter parameters utilizing a disposable concentric needle have been already done for the most often tested muscles. Jitter, expressed as the mean consecutive difference (MCD), was measured in the *Tibialis Anterior* (TA), not routinely tested muscle. Jitter measurement was taken using the intramuscular microaxonal stimulation technique in 32 healthy subjects. The mean MCD and the mean MCD of the 27th value from the 32 subjects had a normal distribution and were 19.79 ± 2.72 μs and 26.88 ± 3.56 μs, respectively. The suggested limit for the mean MCD is ≥ 26 μs and for the individual values is > 34 μs.

## Introduction

Neuromuscular jitter, assessed by single-fiber electromyography (SFEMG), is the variation in time intervals between pairs of single fiber action potentials (SFAP) acquired with voluntary activation or the variation in time observed between stimulus and evoked SFAPs in response to nerve stimulation (s-jitter). Jitter is a sensitive indicator of neuromuscular junction (NMJ) function, and it is handy in examining individuals with suspected NMJ dysfunction, as in myasthenia gravis (MG). Because of the great concern over infection transmission, disposable concentric needle electrodes (CNE) are being used for jitter analysis (1–9) instead of the reusable and expensive single fiber electrode. The term “jitter recording with CNE” is more appropriate than SFEMG because the signal obtained by CNE recording does not always represent one SFAP but rather the summation of many. The term “apparent SFAP” (ASFAP) (3) is preferred instead. The most commonly studied limb muscle for jitter analysis is the *Extensor Digitorum* (ED). Still, it could be affected by C7 radiculopathy presenting active denervation or chronic reinnervation, which increases the jitter values (10). Jitter analysis in limb muscles is augmenting importance due to the massive increase in botulinum neurotoxin (BoNT) injections in the facial muscles, undermining the results (11). This study aimed to determine the reference values of the jitter parameters in the *Tibialis Anterior* (TA) muscle using intramuscular microaxonal stimulation and by recording with disposable CNE.

## Methods

This study was in accord with the Helsinki Declaration of 1975 and approved by the ethics committee of the Faculdade de Medicina de São José do Rio Preto, São Paulo, Brazil, where the SFEMG studies were performed.

### Subjects

Thirty-two adult patients of both sexes were recruited and invited to participate in the study. They were selected from the State Medical School (FAMERP). None had been diagnosed with a neuromuscular disorder, diabetes, injury affecting L5 root, sciatic or fibular nerves or were taking verapamil or amlodipine - calcium channel blockers medications that could increase jitter (12). The temperature was measured on the anterior leg and kept above 30°C.

### Single-fiber electromyography

The SFEMG test was done at the Neuromuscular Investigation Laboratory (LIN) at the FAMERP by the first author (JAK). The electrical activation technique measured jitter parameters in the TA muscle (details below). A Natus electrodiagnostic machine with UltraPro™ S100 Elite software in Viking^®^ display (*Neurodiagnostic System, Middleton, WI, USA*) was used in all subjects. The recordings were performed using a CNE 25 mm × 30 G with a recording area of 0.020 mm^2^ (*Dantec*^®^ *DCN, Natus Manufacturing Limited, Ireland*). The CNE used was approved by the Brazilian Agency for Health Surveillance (ANVISA). An amplitude detection algorithm was used to record and analyze time variations. In all cases, qualitative needle electromyography (EMG) was done in the TA muscle before starting the jitter measurement to exclude active denervation (fibrillation and positive-wave potentials) or chronic reinnervation based on the motor unit action potentials (MUAP) with increased amplitude and duration, which is well-known to increase the jitter (10). If the TA muscle presented any abnormalities described, it was discarded.

**Figure 1:**
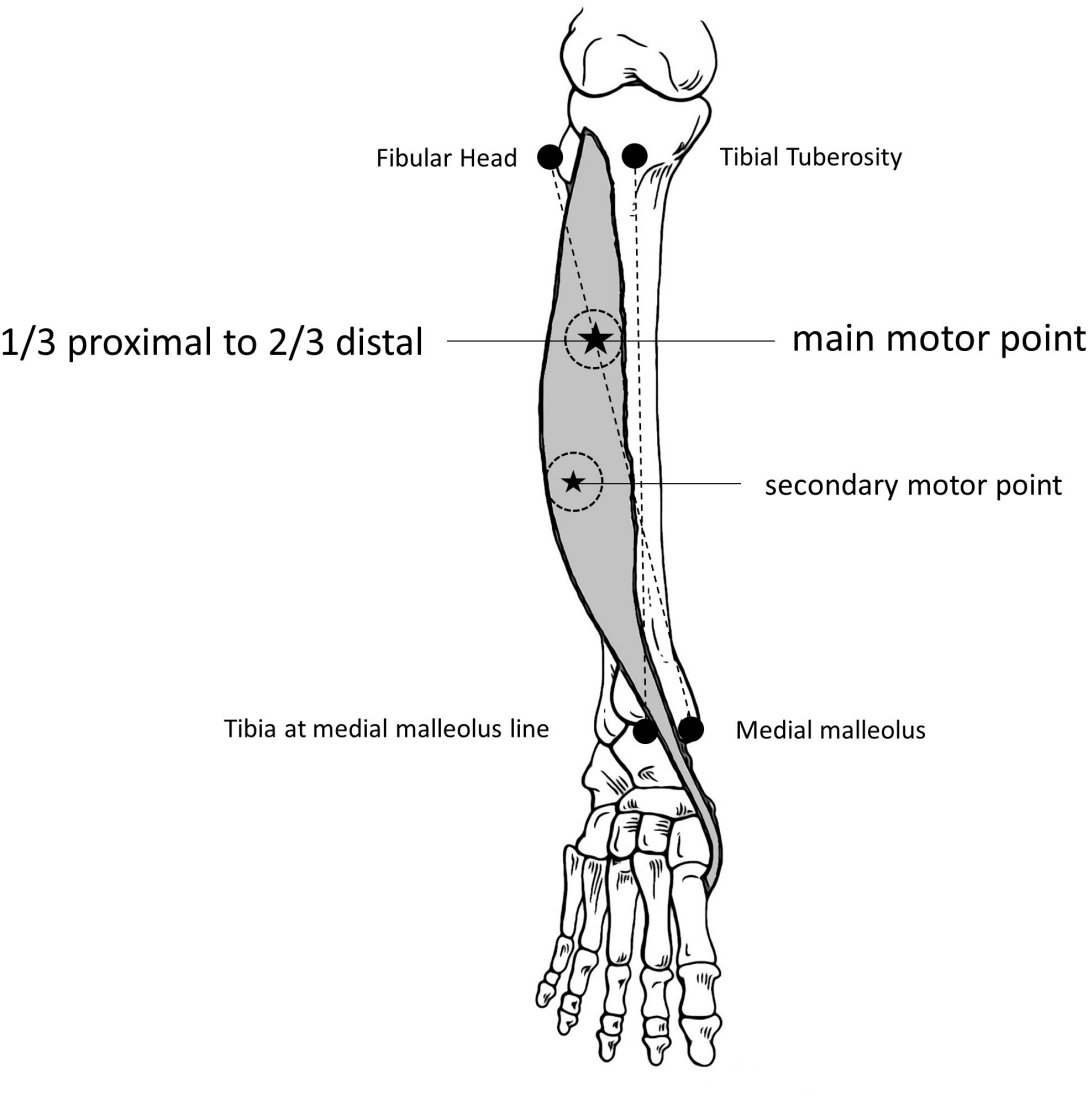
*Tibialis Anterior* muscle anatomical markers for the motor endplate (motor point). See the main and the secondary motor points, where the monopolar stimulation needle should be inserted, and the 2 to 3 cm around, where the concentric needle will be inserted (twitching muscle).

**Figure 2:**
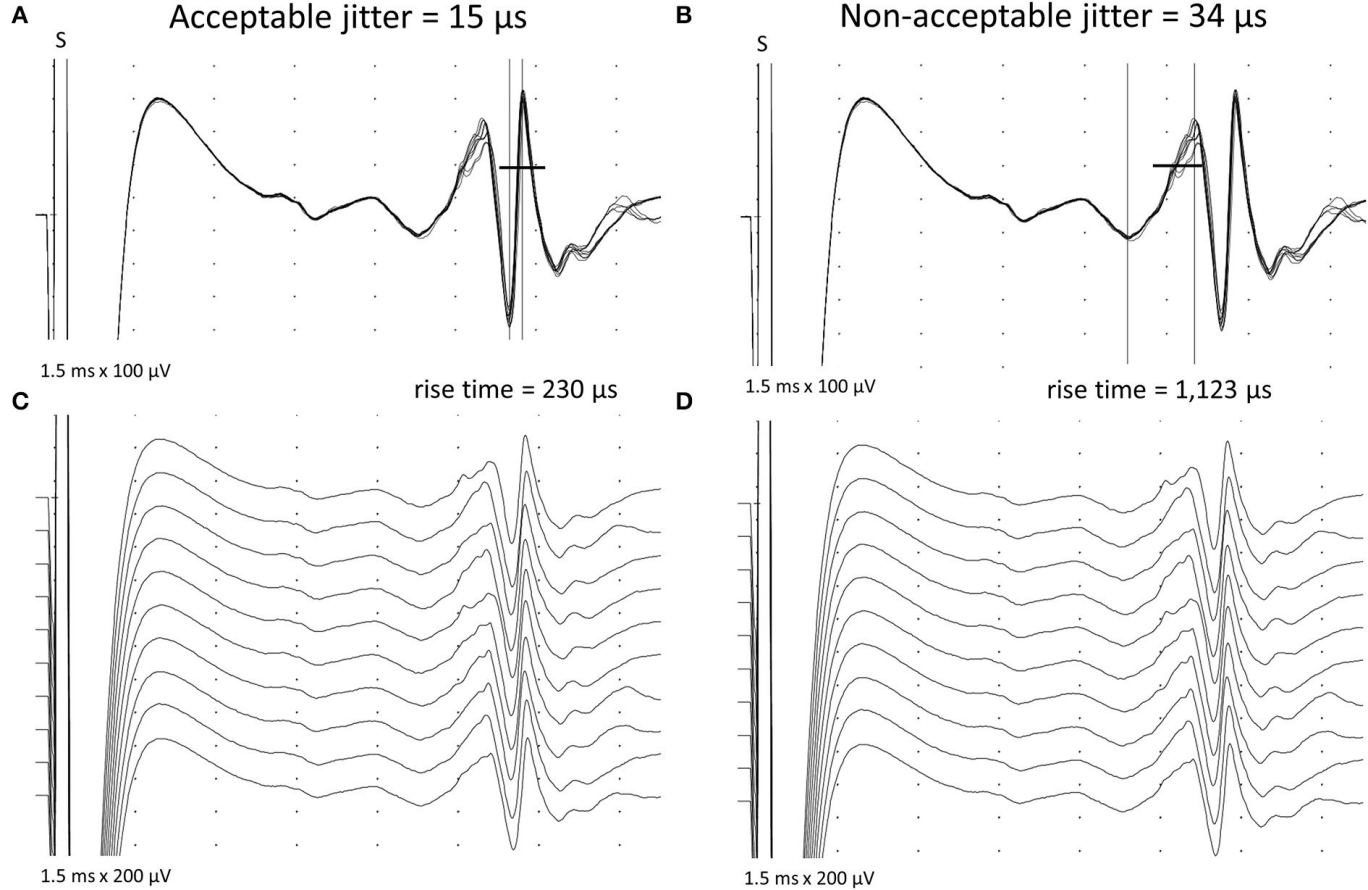
Acceptable and non-acceptable apparent single-fiber action potentials are shown after stimulation jitter (S = stimulus) in the *Tibialis Anterior* muscle. **(A)** Acceptable spikes have a fast-rising phase (less than 300 μs) without notches or shoulders, a well-defined peak, and a constant shape at consecutive discharges **(C)**. Note the parallel negative-going deflection on the acceptable spikes on superimposed traces **(A)**. In **(B)** and **(D)**, non-acceptable spikes with slow rising phase and non-parallel depolarizing negative lines, giving a false increased jitter.

The jitter measurement was performed by intramuscular electrical stimulation delivered by a disposable monopolar needle electrode (cathode), 25 × 36 mm, and with 28 G (*Ambu*^®^*, Neuroline, Malaysia*) inserted near the endplate motor zone (motor point) and positioned at a right angle to the course of the muscle fibers (13). For the reference electrode (anode), an adhesive electrode was used about 1–2 cm away from the cathode, usually proximally. The main motor endplate zone for the TA muscle is relatively undisputed and could be reached in two ways: first, ~1/3 distal to the tibial tuberosity (14); second, by tracing a line between the fibular head to the medial malleolus and marking the limit of the proximal third and the distal two-thirds (Figure 1). However, two endplate motor zones are found: one “classical” and large, as described above, and another smaller, distal, and lateral (15). Stimulation at 10 Hz was delivered as rectangular pulses of 0.05 ms duration, and the intensity was adjusted to produce a slight twitch of the muscle. In general, this could be achieved at about 1–2 mA. The CNE was inserted into the muscle's twitching part, observed 1–3 cm away from the cathode in any direction, and gently positioned to record clearly defined ASFAPs. For each ASFAP suitable for the analysis, the stimulation intensity was finely adjusted to avoid submaximal stimulation, giving rise to jitter that was falsely increased with or without impulse block (16). Maximum stimulation was achieved with no further ASFAP latency reduction (latency stability), frequently associated with new ASFAPs recruitment, and subliminal to the first one. Others have already studied and defined the standard method for the electrical activation (7, 17–20). Acceptable ASFAPs should have a fast-rising phase (<300 μs) without notches or shoulders and have a well-defined peak. The shape should be constant at consecutive discharges and is best seen when 5–10 traces are superimposed (21). The negative-going deflection of the waveforms should be parallel on superimposed traces (Figure 2). A minimum of 50 and an ideal 100 consecutive traces should be recorded for jitter analysis. The low-frequency filter was set from 500 Hz to 1 kHz to suppress distant muscle fiber activity (1, 2, 13). The CNE should be inserted in three to five sites in the muscle alternating with the readjustments of the stimulating electrode to get different ASFAPs; in all the cases, 30 different ASFAPs that were measured in each subject jitter were expressed as the mean of mean consecutive differences (MCD). The mean interpotential interval was calculated as the latency between the stimulus and the response. In each sequence, the analysis should start after about 1 s of stimulation (10 ASFAPs) to exclude the brief initial shortening of latency of the responses to the first few stimuli (22). We excluded jitter values of less than five microseconds as they reflected a direct muscle fiber stimulation (no jitter), and care was taken to avoid “axon-reflex” and f-waves (16). The time spent on this technique was about 30 min. The technique is well-tolerated. Fiber density is not calculated in CNE studies (9).

### Statistics

The Kolmogorov-Smirnov (KS), Anderson-Darling (AD), and Shapiro-Wilk (SW) normality tests were used for the mean MCD (32 values), the mean of the 27th highest MCD (32 values), and the individual MCD (960 values). The mean and standard deviation (SD) values were calculated for data with normal distribution, and the upper limit of normality was calculated as the mean plus 2 SDs. The median and percentiles values were calculated for data with the non-Gaussian distribution, and the upper limit of normality was calculated for the 97.5% highest value. To measure the linear association between two variables, a correlation of r (R-Sq.) was used, with *r* = 1 (R-Sq. = 1) or 100% representing the perfect linear association and *r* = 0 (R-Sq. = 0) representing no association between the variables. The R-Sq was calculated between the mean MCD values and age, MUAP amplitude, and mean interpotential interval values. The software Minitab^®^ was used for statistical evaluations.

## Results

### Patients

Of the 32 patients, 16 were men and 16 were women. The mean, SD, and range of age are shown in Table 1. The correlation coefficient (R-Sq) between the 32 mean MCD values and age is shown in Table 1, with no significant association between the variables.

**Table 1:** Data from the CNE reference s-jitter parameters for the *Tibialis Anterior* muscle in 32 subjects.

**Variables**	**n**	**Gaussian**	**Correlation**	**Median**	**Mean**	**SD**	**Min**	**Max**	**97. 5%**	**+2SD**	**Limit**
Age	32	–	–		40.9	12.6	21	60	–	–	–
Male	16	–	–		–	–	–	–	–	–	–
Female	16	–	–		–	–	–	–	–	–	–
Mean MCD	32	yes	–		19.79 μs	2.72	14.69	24.73	–	25.22	≥26
Mean 27th MCD	32	yes	–		26.88 μs	3.56	17.9	33.4	–	34	>34
All MCD	960	no	–	19.2 μs			5.20	45.7	32.33	–	≥33
Mean MIPI	32	–	–		8.34 ms	1.76	5.77	12.46	–	–	–
Mean MUAP	32	–	–		1,938 μV	–	1,256	3,438	–	–	–
*R*^2^ MCD vs. age	32	–	0.0903		–	–	–	–	–	–	–
*R*^2^ MCD vs. MUAP	30	–	0.1568		–	–	–	–	–	–	–
*R*^2^ MCD vs. MIPI	32	–	0.1927		–	–	–	–	–	–	–

*CNE, concentric needle electrode; Max, maximum; MCD, mean consecutive difference; Min, minimum; MIPI, mean interpotential interval (latency); MUAP, motor unit action potential (amplitude); n, number; NS, not significant; R^2^, coefficient of determination; s-jitter, stimulated jitter; SD, standard deviation. Suggested limits are in bold*.

### Jitter

The mean, SD, and range of the MCD (latencies) are shown in Table 1. The mean jitter was analyzed according to a previously described method, i.e., calculating the mean MCD of 30 ASFAP potentials for s-jitter (13). The 32 mean MCD and the 32 mean MCD for the 27th highest values passed in the normality tests (KS, AD, and SW). The calculated mean, SD, range, and the upper limit of normality of the mean MCD are shown in Table 1. The calculated mean, SD, range, and the upper limit of normality for the mean 27th highest MCD value are shown in Table 1. The 960 individual MCD values did not pass the normality tests (KS, AD, and SW). The calculated median, range, and 97.5 percentile are shown in Table 1. The correlation coefficient (*R*^2^) between the 32 mean MCD values and the mean interpotential interval is shown in Table 1, with no significant association between the variables.

## Discussion

### Reference values

We found a reference limit for the MCD value as ≥26 μs and the individual MCD values as >34 μs. We used the 27th MCD value to calculate the upper limit of normality (mean +2SD, normal distribution) of the individual values (outliers), as done in the CNE multicenter study (23). Instead, we could have used the upper 97.5% value (non-normal distribution) of all individual 960 MCD values, but a more statistical bias toward a lower jitter value was expected. When confronted with our findings, the obtained reference limits did not reveal any abnormal mean MCD. For the individual MCD values, there were none with four values abnormals (>10%). There was no significant correlation between the mean MCD and age (9, 21, 23) when jitter is measured with CNE. There was no significant correlation between the mean MCD and the mean interpotential interval (latency). Still, care should be taken to not measure ASFAPs with long latencies (more than 20 ms) and avoid pitfalls, e.g., f-waves (16, 23); our mean observed latency was 8.34 ms. There was no significant correlation between the mean MCD and MUAP amplitude. We could not find any reference values for CNE s-jitter for the TA muscle. For the voluntary activated CNE-jitter, Zambelis and Anagnostou (24) found a mean MCD limit of 41.6 and 59.7 μs for individual MCD values, respectively. For s-jitter using a single fiber electrode, Chang (25) found a mean MCD of 28.5 μs (SD = 9.1 μs), probably with an upper limit of normality of 46.5 μs.

### The importance of the CNE jitter in the TA muscle

In the last two decades, there was an extraordinary increase in the use of BoNT. In those cases, the jitter analysis could be challenging, enlarging the limitation of the test for NMJ disorders in the facial muscles, *Frontalis*, and *Orbicularis Oculi*. Tested facial muscles near the BoNT injections are the most affected in the percent of patients with increased jitter (up to 40%). The amount of jitter could rise to 56.6% above the reference upper limit and persist even after 11 months from the last injection (11). On the other hand, distant muscles were less affected when BoNT was injected into the facial muscles. Only 13.9% of the patients presented with increased jitter, and the rise above the reference limit was 38% and persisted even after 8 months from the last injection (11).

### The CNE jitter in the TA muscle and MG

The TA muscle was chosen as an alternative to evaluate the NMJ because it is involved in MG. It is also easy to do, causes little pain, has relatively parallel fascicles favoring the needle placement, and the patient has their hands free for using, e.g., a smartphone, thus tolerating a more straightforward test. Sometimes, a trick to get fewer multi-spike ASFAPs is to stimulate 1–3 cm more distally to the motor point; doing that, we can have more probability of stimulating fewer motor axons in fewer multi-spike recordings. Another reason for using the TA muscle as an alternative to confirming NMJ dysfunction in MG is the finding that the repetitive nerve stimulation (RNS) in the segment fibular nerve to *Extensor Digitorum Brevis* muscle reveals 23.1% sensitivity and 100% specificity (amplitude), and 33.3% sensitivity and 92.3% specificity (area) for abnormal decrement (≥10%) between the fourth and the first response in all forms of the disease. In the same cases, SFEMG for any muscle was abnormal in 39.2% (26). Oh et al. (27) described MG cases with abnormal RNS found only in the fibular nerve, recorded in the *Extensor Digitorum Brevis* or the TA muscles. These findings strongly suggested that jitter could be measured in the TA muscle in MG suspicion cases. However, we could not find a specific study for the jitter measurement in the TA muscle for patients with MG to show the sensitivity and specificity. A limitation of the jitter measurement in the TA muscle is the high incidence of L5 radiculopathy. However, the ED muscle test could also share a high incidence of C7 radiculopathy. So, it is essential in both cases to exclude active denervation or chronic reinnervation by EMG (10).

### Some technical issues

As the ASFAP represents the summation of SFAP, separate normative data are essential and were already obtained in the ED, *Frontalis*, and *Orbicularis Oculi* muscles for both voluntary and electrical activation (23). The TA muscle was not contemplated in that study. Peak or amplitude levels for the time of markers to measure the CNE jitter received the same results if care is taken when selecting the ASFAPs, as has been already established (16, 21).

## Conclusion

This study has defined reference limits for the CNE s-jitter obtained from 32 healthy subjects in the TA muscle. After the results, we suggest a mean MCD upper limit of ≥26 μs and a limit of >34 μs for individual values (outliers). The CNE has already been a well-established method to measure jitter with the same sensitivity and specificity as the single fiber electrode, which is safe, well-supported, and reliable.

## Data availability statement

The raw data supporting the conclusions of this article will be made available by the authors, without undue reservation.

## Ethics statement

This study was in accordance with the Helsinki Declaration of 1975 and approved by the Ethics Committee of the Faculdade de Medicina de São José do Rio Preto, São Paulo, Brazil, where the SFEMG studies were performed. The patients/participants provided their written informed consent to participate in this study.

## Author contributions

JK designed the study, prepared the protocol, and carried on the single-fiber electromyography exams. CG selected the subjects, tabulated data, and helped in manuscript revision. All authors participated in interpreting data and read and approved the final manuscript.

## Funding

This work was supported by the Fundação de Amparo à Pesquisa do Estado de São Paulo (FAPESP), Brazil (grant number 2022/02291-1).

## Conflict of interest

The authors declare that the research was conducted in the absence of any commercial or financial relationships that could be construed as a potential conflict of interest.

## Publisher's note

All claims expressed in this article are solely those of the authors and do not necessarily represent those of their affiliated organizations, or those of the publisher, the editors and the reviewers. Any product that may be evaluated in this article, or claim that may be made by its manufacturer, is not guaranteed or endorsed by the publisher.

## Abbreviations

AD: Anderson-Darling
ASFAP: apparent single fiber action potential
BoTN: botulinum neurotoxin
CNE: concentric needle electrode
ED: *Extensor Digitorum* muscle
EMG: electromyography
KS: Kolmogorov-Smirnov
MCD: mean consecutive difference
MG: myasthenia gravis
MUAP: motor unit action potential
NMJ: neuromuscular junction
RNS: repetitive nerve stimulation
SD: standard deviation
SFAP: single fiber action potential
SFEMG: single-fiber electromyography
SW: Shapiro-Wilk
TA: *Tibialis Anterior* muscle.
